# Glomerulopathy in patients with distal duplication of chromosome 6p

**DOI:** 10.1186/s12882-016-0246-2

**Published:** 2016-03-21

**Authors:** Augustina Jankauskienė, Magdalena Koczkowska, Anna Bjerre, Joanna Bernaciak, Franz Schaefer, Beata S. Lipska-Ziętkiewicz

**Affiliations:** Vilnius University, Children hospital affiliate of Vilnius university hospital “Santariskiu klinikos”, Santariskiu 4, LT-08406 Vilnius, Lithuania; Department of Biology and Genetics, Medical University of Gdańsk, Dębinki 1, 80211 Gdańsk, Poland; Department of Pediatrics, Oslo University Hospital, Oslo, Norway; Department of Medical Genetics, Institute of Mother and Child, Warsaw, Poland; Division of Pediatric Nephrology, Center for Pediatrics and Adolescent Medicine, Heidelberg, Germany

**Keywords:** Distal chromosome 6p duplication, Focal segmental glomerulosclerosis, Glomerulopathy, *FOXC1*

## Abstract

**Background:**

Duplication of the distal part of chromosome 6p is a rare genetic syndrome. Renal involvement has been reported in the majority of patients, including a wide range of congenital abnormalities of kidney and urinary tract and, occasionally, a proteinuric glomerulopathy.

**Case presentation:**

Here, we report a 13-year-old girl with 6p25.3p22.1 duplication who presented with proteinuria in infancy, was later diagnosed as focal segmental glomerulosclerosis, progressed to end-stage renal disease and was successfully transplanted.

**Conclusion:**

A systematic literature review suggests that 15–20 % of individuals with distal 6p duplication develop progressive proteinuric glomerulopathy. Monitoring of kidney function should be recommended in all cases.

**Electronic supplementary material:**

The online version of this article (doi:10.1186/s12882-016-0246-2) contains supplementary material, which is available to authorized users.

## Background

Trisomy of the distal fragment of chromosome 6p is extremely rare. So far, less than 50 patients have been reported worldwide, each presenting with a distinct region of aberration, unique chromosomal breakpoints and considerable phenotypic variability [1–3]. Nevertheless, a few features appear consistent: pre- and post-natal growth retardation, developmental delay, intellectual disability, dysmorphia, and congenital malformations, most commonly heart, renal and ocular defects (Table 1).

**Table 1:** Clinical features of the distal chromosome 6p duplication syndrome

Perinatal period: IUGR, general hypotonia, feeding problems, pre- and post-natal growth retardation,
Congenital defects: Brain malformations (cerebellal hypothrophy, hypoplastic corpus callosum, hypothalamic hamartoma, hydrocephalus internus) Heart defects (PDA, ASD, VSD, postvalvular (peripheral) pulmonary stenoses, ventricular hypertrophy, tetralogy of Fallot) GI malformations (choanal atresia, anal atresia, diaphragmatic hernia) CAKUT (kidney hypoplasia, kidney aplasia, ectopic kidneys, hydronephrosis, vesico-ureteral reflux, horseshoe kidney, cystic abnormality of Potter)
Craniofacial abnormalities (dysmorphy) Skull (facial asymmetry, craniosynostosis, frontal bossing, flat occiput) Eye (palpebral ptosis, blepharophimosis, hypotelorism, microophtalmia) Ear (low-set, malformed lobes, thick helices, malformed pinnae) Nose (prominent nasal bridge, short nose, tiny nares) Mouth (small, thin lips; long flat philtrum, high arched palate, small pointed chin, micrognathia, gingival hypertrophy)
Neurological signs Developmental delay Intellectual disability (moderate to severe) Seizures Behavioral problems (autism-spectrum disorders, gentle personality, recurrent laughing spells, low frustration tolerance) Vision problems (nystagmus, strabismus, cataracts, microcorneas, myopia, retinal pigmentary dystrophy, persistent hyperplastic primary vitreous with retinal detachment) Hearing loss
Other Proteinuria/glomerulopathy Growth retardation (proportional microsomy) Recurrent infections Immunodeficiency Skin pigmentary anomalies, capillary hemangiomas

*PDA* patent ductus arteriosus, *ASD* atrial septal defect, *VSD* ventricular septal defect

## Case report

The Central European descent girl with a complex genomic disorder involving chromosome 6p presented with persistent proteinuria from the age of 9 months. Family history was unremarkable. Birth weight, length and head circumference were below the 3^rd^ centile. General hypotonia and failure to thrive prompted a detailed evaluation, which revealed dysmorphia and multiple congenital defects, including corpus callosum hypoplasia, moderate internal hydrocephalus, brain asymmetry, subvalvular pulmonary stenosis and partial synostosis of 1^st^ and 2^nd^ ribs. Distinctive facial features: craniosynostosis, facial asymmetry, high broad front with frontal bossing, microphthalmia with ptosis and blepharophimosis, broad prominent nose bridge with relatively small nose, microstomia, narrow lips and low-set dysplastic ears were observed. Nystagmus, strabismus and severe bilateral hearing impairment developed and developmental milestones were delayed; she was able to sit without support at 14 months and started walking at 39 months of age. At 13 years she still cannot speak and communicates by basic gestures. She understands and follows simple orders.

### Renal involvement

At the age of 9 months the girl was hospitalized because of proteinuria (1.5 g/l). On admission, total serum protein was 63 g/l, albumin 38.5 g/l, cholesterol 6.1 mmol/l, creatinine 49 μmol/l with eGFR 70 ml/min/1.73 m^2^, complement C3 1.54 g/l, C4 0.154 g/l. Serology for prenatal infections was negative. The girl presented no edema or hypertension. Predisolone (15 mg/day) was started but after four weeks proteinuria persisted at 0.75 g/l in spot urine. Predisolone was tapered over two months, and enalapril (1.25 mg/day) was introduced.

Besides persistent proteinuria, in the first 3 years of life the girl presented with at least six febrile episodes of urinary tract infection. No vesicoureteral reflux was revealed by cystography. On ultrasound examination, small kidneys with increased echogenicity and reduced corticomedullary differentiation were noted. Kidney function gradually deteriorated and the child progressed to end-stage kidney disease. Peritoneal dialysis was started at the age of 9.8 years. Six months later kidney transplantation from a living-related donor (mother) was performed and she remains without signs of disease recurrence for 3 years.

### Histopathology studies

Kidney biopsy was performed at age 23 months. Four out of 41 glomeruli were globally obsolescent and two segmentally sclerosed. Immunofluorescence showed weak mesangial positivity for IgM. On electron microscopy, glomerular ultrastructure was generally well preserved, with very few areas exhibiting podocyte foot process effacement, collapse of capillary tuft, and very limited foci of thinning, splintering and lamellation of the glomeruli basement membrane. Mean thickness of the basement membrane was 203 nm. The diagnosis of focal segmental glomerulosclerosis (FSGS) was made.

### Genetic studies

**Fig. 1:**
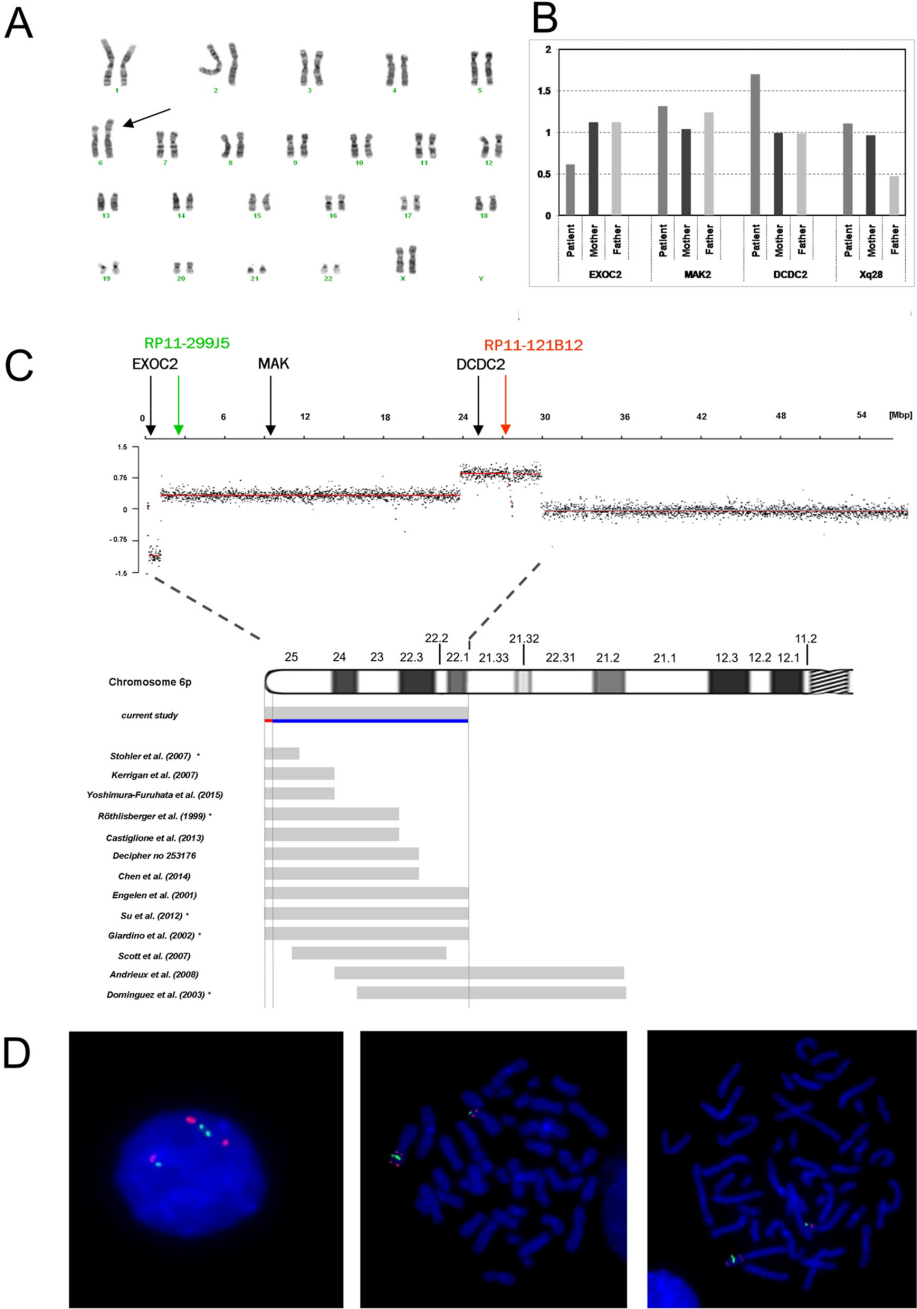
**a-d** Genetic studies of the presented case against reported patients with pure distal 6p duplication. **a** Conventional GTG-banding performed at 550 band level showing additional genetic material at chromosome 6p (arrow). **b** Array CGH analysis. Above chromosome 6 ideogram log2 ratio of patient’s probes are plotted as a function of chromosomal position. Below the chromosome ideogram, mapping of breakpoints of other reported cases is shown. Of these, seven resulted from an insertion of the duplicated 6p fragment on another chromosome and five, including the present case, represent explicitly “pure” 6p duplications resulting from an intrachromosomal rearrangement. Note that for six cases – indicated with black asterisks - determination of the duplicated region was performed on cytogenetic level only, therefore the location of their breakpoints is only approximate. Chromosomal coordinates are presented according to NCBI36/hg18 assembly. The analysis was performed using Human CGH 3x720K Whole-Genome Tiling v3.0 Array (*NimbleGen, Roche*), scanned with MS200 Microarray Scanner (*NimbleGen, Roche*) and analyzed using Nexus Copy Number Software 7.1 (*BioDiscovery*). **c** Quantitative real-time PCR (qPCR) validation of array CGH. Dosage of the three genes localized in region of rearrangement: *EXOC2*, *MAK* and *DCD2* (their *loci* indicated by arrows) relative to two reference genes: *GPR15* (3q11.2) and *ERMP1* (9p24.1) and normalized to reference DNA. Patient and her parents DNA were analyzed against a reference control DNA. All samples were run in triplicate. The diploid (two copies of a gene) samples have the normalized ratio = 1 (gray-colored band); whereas the normalized ratio of 1.5 and 0.5 refers to triploid and monoploid samples respectively. **d** FISH FISH analysis was performed using standard procedures with the bacterial artificial chromosome (BAC) clones specific for the aberration’s regions. The chromosome 6 breakpoints were mapped to 6p25.2 with BAC clone RP11-299 J5 (green signal) and to 6p22.1 with BAC clone RP11-121B12 (red signal). Left interphase; middle and right – metaphase

Conventional cytogenetic testing showed 46,XX,add(6)(p21), whereas parents exhibited normal karyotypes (Fig. 1a). Array-CGH analysis revealed female hybridization pattern with ~1 Mb terminal deletion at 6p25 encompassing *loci* of seven genes and adjacent interstitial duplication of ~28 Mb consisting of 254 genes (Fig. 1c). The results were confirmed by qPCR (Fig. 1b) and FISH studies (Fig. 1d). Finally, the karyotype was established as follows: 46,XX,dup(6p25p21).ish inv(6)(p25.3)(RP11-299 J5+)(p22.1)(RP11-121B12++).arr[hg18]6p25.3(92,934-1,172,037)x1,6p25.3p22.1(1,172,038-29,167,599)x3.

Mutational screening of 31 glomerulopathy-associated genes using a custom-designed multiplex PCR (*Multiplicom,* Niel, Belgium) revealed no pathogenic mutation in any of the genes tested, *NPHS1*, *NPHS2* and *WT1* included.

## Discussion

In most cases reported, distal trisomy 6p results from an unbalanced inheritance of a parental translocation. The clinical picture of these patients is influenced by the accompanying monosomy of an another chromosomal region. Here, we present an extremely rare case of a pure chromosome 6p duplication resulting from *de novo* reverse duplication (Fig. 1d). A review of the published cases along with database search [1–7] allowed for identification of 12 individuals whose region of duplication was partially overlapping with the region affected in our case (Fig. 1c).

Renal abnormalities have been observed in approximately half of the patients. Congenital abnormalities of the kidney and urinary tract (CAKUT), such as hypoplastic kidneys, renal cysts, vesicoureteral reflux and hydronephrosis have been described [4, 6, 7]. In addition, proteinuria has been reported in eight patients. These included two cases of a pure 6p duplication [5, 7] and six with a duplication resulting from an unbalanced parental translocation (after [4, 7–9]). The presented case is, however, the first to be comprehensively studied for mutations in all known glomerulopathy-associated genes to rule out co-incidence of two genetic entities.

Based on the findings reported to date, we estimate that at least 15–20 % of patients with terminal 6p duplication might develop proteinuria. This may be an underestimate since kidney disease may still be clinically silent at the time when the genetic diagnosis is established. The age at onset of proteinuria was less than three years in 7/9 subjects including the present case; the remaining two developed glomerular failure before the age of ten years. Six patients underwent kidney biopsy. Of these, three were diagnosed with FSGS, while membranous glomerulonephritis, focal mesangial hypercellularity and periglomerular fibrosis was reported each in one case. Five patients underwent kidney transplant at age 4–18 years; so far no disease recurrence has been reported, suggesting an intrinsic renal rather than a systemic abnormality as the cause of glomerulopathy. Resistance to steroids was observed In two treated subjects including the present case.

Giardino et al. [5] proposed 6p22– > 6pter as the most likely localization of the putative *locus* of the causative gene(s). Taking into account breakpoints of a few patients published afterwards including our case, we are able to further narrow the smallest region of the overlap to the 6p25 band. The assigned region comprises 29 protein-coding genes and three miRNAs (Additional file 1: Table S1). None of the established glomerulopathy-associated genes is located within the region of interest. A recent review described candidate genes expected to be involved in phenotypic features of the distal chromosome 6p duplication syndrome [6]. Of these, only one, namely *FOXC1,* was identified as a player in kidney development and functioning. *FOXC1* encodes a transcription factor that, along with Lmx1b, interacts synergistically through a common enhancer site to regulate the expression of podocyte specific genes, including *NPHS2* [10]. Mutations in *FOXC1* have previously been described in patients with Axenfeld-Rieger anomaly and/or glaucoma (OMIM*601090). Haploinsufficiency studies have identified *FOXC1* as a strongly dosage sensitive gene, supporting the hypothesis that *FOXC1* may be pathogenic if overexpressed.

## Conclusions

Our findings reinforce the hypothesis that the proximal 6p region contains gene(s) involved not only in kidney development but also in postnatal podocyte function. We recommend monitoring of kidney morphology and function in all patients with constitutive distal 6p duplication. Conversely, in patients with features of CAKUT and/or SRNS/FSGS who have associated syndromic disease array-CGH evaluation for chromosome 6p duplication should be performed.

## Additional file

Additional file 1: List of the genes in the smallest region of overlap in patients with constitutional distal chromosome 6p duplication presenting with glomerulopathy. (PDF 457 kb)

## References

[1] Vulto-van Silfhout AT, van Ravenswaaij CM, Hehir-Kwa JY, Verwiel ET, Dirks R, van Vooren S, Schinzel A, de Vries BB, de Leeuw N (2013). An update on ECARUCA, the European Cytogeneticists Association Register of Unbalanced Chromosome Aberrations. Eur J Med Genet.

[2] Firth HV, Richards SM, Bevan AP, Clayton S, Corpas M, Rajan D, Van Vooren S, Moreau Y, Pettett RM, Carter NPDECIPHER (2006). Database of Chromosomal Imbalance and Phenotype in Humans Using Ensembl Resources. Am J Hum Genet.

[3] Dominquez MG, Wong-Ley LE, Rivera H, Vasquez AL, Ramos AL, Sanchez-Urbina R, Morales JA, Figuera LE (2003). Pure partial trisomy 6p due to a familial insertion (16;6)(p12;p21.2p23). Ann Genet.

[4] Engelen JJ, Marcelis CL, Alofs MG, Loneus WH, Pulles-Heintzberger CF, Hamers AJ (2001). De novo “pure” partial trisomy (6)(p22.1 → pter) in a chromosome 15 with an enlarged satellite, identified by microdissection. Am J Med Genet.

[5] Giardino D, Finelli P, Caufin D, Gottardi G, Lo Vasco R, Turolla L, Larizza L (2002). Pure 6p22-pter trisomic patient: refined FISH characterization and genotype - phenotype correlation. Am J Med Genet.

[6] Castiglione A, Guaran V, Astolfi L, Orioli E, Zeri G, Gemmati D, Bovo R, Montaldi A, Alghisi A, Martini A (2013). Karyotype-phenotype correlation in partial trisomies of the short arm of chromosome 6: a family case report and review of the literature. Cytogenet Genome Res.

[7] Yoshimura-Furuhata M, Nishimura-Tadaki A, Amano Y, Ehara T, Hamasaki Y, Muramatsu M, Shishido S, Aikawa A, Hamada R, Ishikura K, Hataya H, Hidaka Y, Noda S, Koike K, Wakui K, Fukushima Y, Matsumoto N, Awazu M, Miyake N, Kosho T (2015). Renal complications in 6p duplication syndrome: Microarray-based investigation of the candidate gene(s) for the development of congenital anomalies of the kidney and urinary tract (CAKUT) and focal segmental glomerular sclerosis (FSGS). Am J Med Genet A.

[8] Pierpont ME, Hentges AS, Gears LJ, Hirsch B, Sinaiko A (2000). Unbalanced 4;6 translocation and progressive renal disease. Am J Med Genet.

[9] Belligni EF, Biamino E, Molinatto C, Messa J, Pierluigi M, Faravelli F, Zuffardi O, Ferrero GB, Silengo MC (2009). Subtelomeric FISH analysis in 76 patients with syndromic developmental delay/intellectual disability. Ital J Pediatr.

[10] He B, Ebarasi L, Zhao Z, Guo J, Ojala JR, Hultenby K, De Val S, Betsholtz C, Tryggvason K (2014). Lmx1b and FoxC combinatorially regulate podocin expression in podocytes. J Am Soc Nephrol.

