# Erratum to: Glomerulopathy in patients with distal duplication of chromosome 6p

**DOI:** 10.1186/s12882-016-0255-1

**Published:** 2016-04-26

**Authors:** Augustina Jankauskienė, Magdalena Koczkowska, Anna Bjerre, Joanna Bernaciak, Franz Schaefer, Beata S. Lipska-Ziętkiewicz

**Affiliations:** Vilnius University, Children hospital affiliate of Vilnius university hospital “Santariskiu klinikos”, Santariskiu 4, Vilnius, LT 08406 Lithuania; Department of Biology and Genetics, Medical University of Gdańsk, Dębinki 1, Gdańsk, 80211 Poland; Department of Pediatrics, Oslo University Hospital, Oslo, Norway; Department of Medical Genetics, Institute of Mother and Child, Warsaw, Poland; Division of Pediatric Nephrology, Center for Pediatrics and Adolescent Medicine, Heidelberg, Germany

## Erratum

Following publication of the original article in *BMC Nephrology* [1], we would like to alert the reader that the displayed grant number (011/01/D/NZ2/01600) is incorrect and should read as 2011/01/D/NZ2/01600.

The original article has been updated with the correct grant number. The publisher apologizes for the inconvenience this may have caused.

## References

[1] Jankauskienė A (2016). Glomerulopathy in patients with distal duplication of chromosome 6p. BMC Nephrol.

