# Supplementary material for: Glomerulopathy in patients with distal duplication of chromosome 6p
Source: BMC Nephrol. 2016 Mar 21;17:32. doi: 10.1186/s12882-016-0246-2 (PMC4802583; doi:10.1186/s12882-016-0246-2)
Supplement: Additional file 1: — List of the genes in the smallest region of overlap in patients with constitutional distal chromosome 6p duplication presenting with glomerulopathy. (PDF 457 kb) [file 12882_2016_246_MOESM1_ESM.pdf]

**Supplementary Table 1.**

**List of the genes present in the smallest region of overlap in patients with constitutional distal chromosome 6p duplication presenting with glomerulopathy.**

The entries are based on information collected at OMIM (http://www.ncbi.nlm.nih.gov/omim/), NCBI GENE (http://www.ncbi.nlm.nih.gov/gene/) and Nexus Copy Number Software ver 7.5 (Biodiscovery, Hawthorne, CA) databases.

Chromosomal coordinates are given according to the UCSC genome annotation database for the Mar. 2006 GenBank freeze assembled by NCBI (hg18, Build 36.1).

| Gene | Name | OMIM ID | Chromosomal coordinates | Function of the encoded protein. |
|---|---|---|---|---|
| **Protein-coding genes with RefSeq.** | | | | |
| ***FOXQ1*** | forkhead box Q1 | *612788 | 1257674-1259993 | Transcription factor involved in embryonic development, cell cycle regulation, tissue-specific gene expression, cell signaling, and tumorigenesis |
| ***FOXF2*** | forkhead box F2 | *603250 | 1335067-1340831 | Transcription factor involved in embryonic development (limbs, gastrointestinal track, genitalia, epithelial to mesenchymal transition, extracellular matrix organization) |
| ***FOXC1*** | forkhead box C1 | *601090 #602482 #601631 | 1555679-1559128 | Transcription factor involved in embryonic development (ocular, cardiovascular and neuronal in particular) tumorigenesis and epithelial–mesenchymal transition. **Genotype -phenotype**: Axenfeld-Rieger syndrome, type 3; Iridogonidys-genesis, type 1 |
| ***GMDS*** | GDP-mannose 4,6-dehydratase | *602884 | 1569033-2190867 | EC 4.2.1.47. Catalyzes the conversion of GDP-mannose to GDP-4-keto-6-deoxymannose, the first step in the synthesis of GDP-fucose from GDP-mannose, using NADP+ as a cofactor. |
| ***MYLK4*** | myosin light chain kinase family, member 4 | n/a | 2608861-2696153 | Protein phosphorylation (Serine/Threonine protein kinase) |
| ***WRNIP1*** | Werner helicase interacting protein 1 | *608196 | 2710664-2730978 | Replication factor that interacts with WRN, the protein that is defective in Werner syndrome. Accumulates at sites of DNA damage by interacting with polyubiquinated proteins and also binds to DNA polymerase delta. |
| ***SERPINB1*** | serpin peptidase inhibitor, clade B (ovalbumin), member 1 | *130135 | 2777564-2787282 | Monocyte/neutrophil elastase inhibitor, a member of the serpin (serine proteinase inhibitor) family of proteinase inhibitors |
| ***SERPINB6*** | serpin peptidase inhibitor, clade B (ovalbumin), member 6 | *173321 #613453 | 2893391-2917398 | Member of the serpin (serine proteinase inhibitor) family, originally discovered as a placental thrombin inhibitor. Plays an important role in the inner ear in the protection against leakage of lysosomal content during stress. **Genotype-phenotype**: Deafness, autosomal recessive 91 |
| ***SERPINB9*** | serpin peptidase inhibitor, clade B (ovalbumin), member 9 | *601799 | 2832498-2848545 | Member of the serpin (serine proteinase inhibitor) family. Endogenous inhibitor of caspase-1. |
| ***NQO2*** | NAD(P)H dehydrogenase, quinone 2 | *160998 #114480 | 2945048-2965109 | EC 1.10.99.2. NAD(P)H:quinone acceptor oxidoreductase-2 , a member of the thioredoxin family of enzymes. Catalyzes the 2-electron reduction of various quinones, redox dyes, and the vitamin K menadione **Genotype-phenotype**: susceptibility to breast cancer |
| ***RIPK1*** | receptor (TNFRSF)-interacting serine-threonine kinase 1 | *603453 | 3022056-3060420 | A key signaling molecule in the programmed necrosis pathway, which plays important roles in development, tissue damage response, and antiviral immunity. |
| ***BPHL*** | biphenyl hydrolase-like | *603156 | 3063924-3098431 | A hydrolytic enzyme that contains a serine residue in their active site. |
| ***TUBB2A*** | tubulin, beta class IIa | *615101 # 615763 | 3098900-3102782 | Structural element of microtubules. Plays important role in mitosis, intracellular transport, neuron morphology, and ciliary and flagellar motility. Highest expression in brain, followed by gastrointestinal track and kidney. **Genotype-phenotype**: Cortical dysplasia, complex, with other brain malformations 5 |
| ***TUBB2B*** | tubulin, beta class IIb | *612850 #610031 | 3169493-3172967 | Structural element of microtubules. Plays important role in mitosis, intracellular transport, neuron morphology, and ciliary and flagellar motility. Expression predominantly in central and peripheral nervous system during neuronal migration and differentiation. **Genotype-phenotype**: Polymicrogyria, symmetric or asymmetric |
| ***PSMG4*** | proteasome (prosome, macropain) assembly chaperone 4 | n/a | 3204160-3213299 | Chaperone protein which promotes assembly of the 20S proteasome |

| Gene | Name | OMIM ID | Chromosomal coordinates | Function of the encoded protein. |
|---|---|---|---|---|
| ***SLC22A23*** | solute carrier family 22, member 23 | *611697 | 3214205-3401792 | Member of a large family of transmembrane proteins that function as uniporters, symporters, and antiporters to transport organic ions across cell membranes |
| ***PXDC1*** | PX domain-containing protein 1 | n/a | 3667834-3697245 | A protein with one phox-homology domain of unknown function. |
| ***FAM50B*** | family with sequence similarity 50, member B | *614686 | 3794630-3796550 | A functional retrotransposon on chromosome 6 derived from the FAM50A gene on X chromosome. Its expression is regulated by the mechanism of parental (paternal) imprinting. The function of the encoded protein remains unknown, however its expression is observed during spermato-genesis and tumorigenesis. |
| ***PRPF4B*** | pre-mRNA processing factor 4B | *602338 | 3966567-4010216 | The protein encoded is involved in pre-mRNA splicing and in signal transduction. Regarded as a CDK-like kinase (Clk) with homology to mitogen-activated protein kinases (MAPKs). |
| ***FAM217A*** | family with sequence similarity 217, member A | n/a | 4013591-4024456 | Encodes a protein of unknown function. Is most abundantly expressed in tissues rich in highly ciliated cells, such as olfactory sensory neurons, and is predicted to be important to cilia. |
| ***ECI2*** | peroxisomal D3,D2 enoyl-CoA isomerase | *608024 | 4060925-4080830 | The protein encoded is a key mitochondrial enzyme involved in beta-oxidation of unsaturated fatty acids. |
| ***CDYL*** | chromodomain protein, Y-like | *603778 | 4651391-4900777 | Autosomal homolog of chromodomain Y. Similarly, it possess a chromo-domain, a motif implicated in chromatin binding and gene suppression, and a catalytic domain believed to be involved in histone acetylation. |
| ***RPP40*** | ribonuclease P/MRP 40kDa subunit | *606117 | 4940278-4949296 | An element of complex forming Ribonuclease P (RNase P) that removes the 5-prime leader sequences from precursor tRNA molecules. |
| ***PPP1R3G*** | protein phosphatase 1, regulato-ry subunit 3G | n/a | 5030718-5032454 | A glycogen-targeting subunit that in combination with protein phosphatase 1 controls glycogenesis in different organs. |
| ***LYRM4*** | LYR motif containing 4 | *613311<br>#615595 | 5053651-5206182 | A mitochondrial protein, member of the ISCU/NFS1/LYRM4 complex responsible for the biogenesis of Fe-S (iron- sulphur) clusters. It interacts directly with the complex activator frataxin.<br>**Genotype-phenotype**: Combined oxidative phosphorylation deficiency 19; susceptibility to cognitive defect in schizophrenia |
| ***FARS2*** | mitochondrial phenylalanyl-tRNA synthetase 2 | *611592<br>#614946 | 5206582-5716815 | A member of the class II aminoacyl-tRNA synthetases that catalyze attachment of phenylalanine to its cognate tRNA. |
| ***NRN1*** | neuritin 1 | *607409 | 5943230-5952837 | A glycosylphosphatidylinisotol (GPI)-anchored neuronal protein that functions extracellularly to promote neurite outgrowth and branching. It is a glutamate and neurotrophin receptor target gene. |
| ***F13A1*** | coagulation factor XIII, A1 subunit | *134570<br>#613255<br>#608446<br>#188050 | 6089309-6265923 | EC 2.3.2.13. A subunit of factor XIII, the last zymogen to become activated in the blood coagulation cascade.<br>**Genotype-phenotype**: factor XIII deficiency (bleeding disorder); protection against myocardial infarction, protection against venous thrombosis) |
| ***LY86*** | lymphocyte antigen 86 | *605241 | 6533932-6600215 | A leucine rich repeat (LRR)-bearing molecule that transmits an activation signal leading to massive B-cell proliferation as well as resistance against apoptosis. |
| ***Non-coding mRNA molecules*** | | | | |
| ***MIR6720*** | microRNA 6720 | n/a | 1335547-1335645 | no data |
| ***MIR4645*** | microRNA 4645 | n/a | 2799263-2799340 | no data |
| ***MIR3691*** | microRNA 3691 | n/a | 5093465-5093555 | no data |
| ***LINC01011*** | long intergenic non-protein coding RNA 1011 | n/a | 2933199-2936404 | no data |
| ***GMDS-AS1*** | GMDS antisense RNA 1 | n/a | 2190985-2358824 | no data |
| ***LY86-AS1*** | LY86 antisense RNA 1 | n/a | 6291696-6568058 | no data |

| Gene | Name | OMIM ID | Chromosomal coordinates | Function of the encoded protein. |
|---|---|---|---|---|
| ***Open reading frames*** | | | | |
| ***C6orf195*** | chromosome 6 open reading frame 195 | n/a | 2567970-2580297 | Uncharacterized protein |
| ***C6orf201*** | chromosome 6 open reading frame 201 | n/a | 4024438-4075998 | Uncharacterized protein |
| ***SERPINB9P1*** | serpin peptidase inhibitor, clade B member 9, pseudogene 1 | n/a | 2799889-2821743 | pseudogene |
| ***HTATSF1P2*** | HIV-1 Tat specific factor 1 pseudogene 2 | n/a | 2965388-2970004 | pseudogene |
